# Updates on Glycaemic Control Strategies: A Range of Opportunities after Total Pancreatectomy

**DOI:** 10.3390/jcm12093306

**Published:** 2023-05-06

**Authors:** Silvia Pieralice, Alessandro Coppola, Ernesto Maddaloni

**Affiliations:** 1 Department of Experimental Medicine, Sapienza University of Rome, Viale Regina Elena 324, 00161 Rome, Italy; 2 Department of Surgery, Sapienza University of Rome, 00161 Rome, Italy

In the past, indications for total pancreatectomy (TP) were rare, with several concerns about patients’ postoperative quality of life due to exocrine and endocrine post-pancreatectomy management [1].

Recently, the number of patients undergoing TP has increased. Previously, TP was performed only in patients with chronic pancreatitis or for intraoperative findings of persistent cancer infiltration at intraoperative frozen sections. Currently, these classic indications have been enlarged by TPs performed for the treatment of intraductal papillary mucinous neoplasm (IPMN) [2] or for pancreatic resections with extensive arterial vascular resections and reconstruction [3].

In addition, postoperative pancreatic fistula represents the Achilles’ heel of pancreatic surgery [4]; TPs have been proposed as a possible surgical treatment to avoid this complication in patients with a high perioperative risk of mortality [5].

Although numerous articles have reported on the positive quality of life that patients can achieve after TP [6], some doubts still remain regarding glycemic control in these patients. Similar to other HPB settings [7], patient candidates for TP should be discussed in a multidisciplinary meeting and treated by multidisciplinary teams.

In order to safely propose patients for TP, it is also mandatory for the surgical community to be updated on newly available glycemic control methods.

The primary aim of diabetes management is to achieve optimal glycemic control in the long term in order to prevent or delay diabetes-related complications. Although intensive insulin therapy represents the gold standard regimen for subjects with insulin deficiency [8], this approach requires continuous self-decisions and the exposure of people with diabetes to the risk of iatrogenic hypoglycemia, resulting in the high burden of diabetes management and making it difficult to achieve the goal of glycated hemoglobin (HbA1c) below 7% (53 mmol/mol) [8]) and with other clinical targets [9], as recommended by international guidelines. Thus, reducing the risk of hypoglycemia is a key concurrent goal of diabetes therapy, often contrasting with therapies aiming to maintain strict glycemic control.

However, during recent decades, diabetes technology has rapidly improved, particularly in the areas of insulin pumps and continuous glucose monitoring (CGM), reducing blood glucose fluctuations in daily living activities and ameliorating a perceived quality of life [10].

Continuous subcutaneous insulin infusion (CSII) systems are programmable pumps that continuously deliver rapid-acting insulin into the subcutaneous tissue. Compared to multiple daily injections (MDI), CSII improves flexibility in terms of prandial bolusing and physical activity while also improving glycemic control during the early morning and fasting, illness, and stressful periods.

On the other hand, the CGM system consists of a subcutaneous sensor that measures the glucose concentration continuously in the interstitial fluid, providing near real-time glucose measurements. This approach allows subjects to monitor their glucose levels and trend, demonstrating beneficial qualities in both type 1 and type 2 diabetes irrespective of treatment regimens.

Novel combined systems have been launched in recent decades with to integrate CSII and CGM technologies. The first integrated systems (Low Glucose Suspend systems, LGS) were able to automatically and temporarily stop insulin delivery when blood glucose levels reached the hypoglycemia threshold (<70 mg/dL). Soon after, the Predictive Low Glucose Suspend system (PLGS) went beyond LGS, improving this technology by predicting hypoglycaemia and suspending insulin delivery before this adverse event could occur. Although both LGS and PLGS have proven to reduce the frequency and severity of hypoglycaemia, these systems did not help the prevention of hyperglycemic spikes.

Afterward, more complex glucose-responsive insulin delivery algorithms were developed, to automatically address both hypoglycaemia and hyperglycaemia, promoting appropriate glycemic control during the day. Hybrid closed loops (HCL) and advanced hybrid closed loops (AHCL) are technology-advanced insulin pumps characterized by the coexistence of algorithm-driven automated insulin delivery, based on glucose sensor values, with manual mealtime boluses. These technologies consist of three technologies: a CGM sensor that transmits continuous glycaemic values to an algorithm; an algorithm that analyzes CGM data and calculates the insulin required to prevent hyperglycemic and hypoglycemic events; CSII that delivers insulin according to an algorithm [11].

HCL and AHCL systems, known as “artificial pancreas”, modulate insulin delivery continuously and deliver adjunctive small, automated correction boluses when glucose is predicted to rise above the range (AHCL). To date, three main types of closed-loop control algorithms have been used in clinical practice.

The first algorithm was the proportional, integral, derivative (PID) controller. This is an algorithm that treats by directing targeting insulin doses based on the difference between the target glucose at the current point in time (proportional), the rate of variation in glycemic values over time (derivative), and the area under the curve between the glycemia value detected and the glucose levels to be reached (integral) [12]. The second algorithm, known as model predictive control (MPC), is the most widespread type of closed-loop technology. This algorithm is based on a mathematical model that links insulin delivery to glucose excursions. MPC can be dynamic and multi-compartmental, predicting glucose levels while simultaneously adjusting insulin delivery from treatment to target. This system can minimize the impact of insulin absorption delays as well as the impact of meals on blood glucose levels while also accounting for active insulin. 

Lastly, the Fuzzy logic approach regulates insulin infusion rates by applying rules derived from the clinical practice of diabetologists and experts. Furthermore, it is able to suspend insulin delivery when glucose is low or is expected to decrease disproportionately.

Several studies have compared novel artificial pancreas systems and conventional insulin therapy. MPC-based artificial pancreas systems have been shown to improve the percentage of time spent in the euglycemic range better than both traditional insulin therapy and the sensor-augmented pump [13,14]. A study by Pinsker and colleagues also suggested some benefits to the MPC algorithm over the PID algorithm [14].

However, irrespective of the algorithm used, HCL and AHCL have been shown to improve glycemic control, increasing time in the glucose range (70–180 mg/dL) while simultaneously decreasing the rates of hypoglycemic events [15], especially in the absence of prandial glucose excursions. Indeed, the ability to prevent hyperglycemia events from following the consumption of unannounced carbohydrates remains the primary limit of these technologies [16]. This is partly due to the delays in CGM sensing to detect rising glucose levels during meals and partly due to delays in the initiation of insulin action following its infusion, avoiding, in the meantime, potential hypoglycemia, which is secondary to overly aggressive insulin administration.

Because of these considerations, all current commercially available devices require the subject to enter the grams of carbohydrate they have ingested, receiving advice on the “recommended bolus” based on a pre-set prandial insulin-to-carbohydrate ratio and insulin sensitivity factor. However, a recent randomized controlled trial published on Diabetes Care evaluated a fully automated closed-loop control system in the absence of carbohydrate intake announcement, showing an improved time in range (TIR) following an unannounced meal for the algorithm called RocketAP (RCKT) and a novel MPC system with a dedicated bolus priming system to address meal-like disturbances compared with a legacy closed-loop system [17].

In conclusion, the development of new technologies applied to diabetes care allowed a significant improvement in the glycaemic control of people with insulin-dependent diabetes, also reducing the risk of hypoglycemia [18]. By reducing the burden of diabetes, the most novel closed-loop systems could improve quality of life and may be transformative for diabetes care management, ameliorating glycemic outcomes and, consequently, the risk of acute and chronic diabetes-related complications across a broad age range, irrespective of diabetes type, including those with post-pancreatectomy insulin-dependent diabetes. These improvements will likely impact therapeutic decisions when balancing the risks and benefits of different types of pancreatic surgery.
